# Supplementary material for: Influenza A Virus Infection Reactivates Human Endogenous Retroviruses Associated with Modulation of Antiviral Immunity
Source: Viruses. 2022 Jul 21;14(7):1591. doi: 10.3390/v14071591 (PMC9320126; doi:10.3390/v14071591)
Supplement: Supplementary file 1 [file viruses-14-01591-s001.zip › MotifAnalysis.zip!/MotifOutput_SC35M/homerResults/motif5.similar.html]

motif5

## Information for motif5

A
G
T
C
A
G
C
T
T
C
A
G
A
C
G
T
C
T
A
G
A
C
G
T
G
A
T
C
A
C
G
T
C
G
T
A
A
C
T
G
A
G
T
C
C
G
A
T
  
Reverse Opposite:  

C
G
T
A
A
C
T
G
A
G
T
C
A
C
G
T
C
G
T
A
C
T
A
G
G
T
C
A
A
G
T
C
C
G
T
A
A
G
T
C
C
T
G
A
C
T
A
G
  

|  |  |
| --- | --- |
| p-value: | 1e-16 |
| log p-value: | -3.771e+01 |
| Information Content per bp: | 1.826 |
| Number of Target Sequences with motif | 10.0 |
| Percentage of Target Sequences with motif | 24.39% |
| Number of Background Sequences with motif | 136.3 |
| Percentage of Background Sequences with motif | 0.29% |
| Average Position of motif in Targets | 108.8 +/- 49.0bp |
| Average Position of motif in Background | 99.7 +/- 86.9bp |
| Strand Bias (log2 ratio + to - strand density) | 10.0 |
| Multiplicity (# of sites on avg that occur together) | 3.20 |
| Motif File: | file (matrix) reverse opposite |

### Similar de novo motifs found

|  |  |  |  |  |  |  |  |
| --- | --- | --- | --- | --- | --- | --- | --- |
| Rank | Match Score | Redundant Motif | P-value | log P-value | % of Targets | % of Background | Motif file |
| 1 | 0.737 | A C G T C T G A A C G T G C T A A C G T C G T A A C T G A G T C A C G T T G C A | 1e-13 | -30.243640 | 21.95% | 0.40% | motif file (matrix) |
| 2 | 0.686 | A C G T T G C A A C G T A T G C A C G T T C G A A C T G T G C A | 1e-9 | -21.598839 | 29.27% | 2.63% | motif file (matrix) |
